# Humoral immune response and coated or uncoated oxygenators during cardiopulmonary bypass surgery

**DOI:** 10.5830/CVJA-2016-003

**Published:** 2016

**Authors:** Sedat Ozan Karakisi, Şahin Bozok, Şaban Ergene, Gökhan İlhan, Hakan Karamustafa, Nebiye Tufekci, Ayşe Gül Kunt, Erol Şener, İdil Çankaya, Mustafa Kocakulak, Uğur Muşabak, Mustafa Fevzi Sargon

**Affiliations:** Department of Cardiovascular Surgery, Faculty of Medicine, Recep Tayyip Erdogan University, Training and Research Hospital, Rize, Turkey; Department of Cardiovascular Surgery, Faculty of Medicine, Recep Tayyip Erdogan University, Training and Research Hospital, Rize, Turkey; Department of Cardiovascular Surgery, Faculty of Medicine, Recep Tayyip Erdogan University, Training and Research Hospital, Rize, Turkey; Department of Cardiovascular Surgery, Faculty of Medicine, Recep Tayyip Erdogan University, Training and Research Hospital, Rize, Turkey; Department of Cardiovascular Surgery, Faculty of Medicine, Recep Tayyip Erdogan University, Training and Research Hospital, Rize, Turkey; Department of Cardiovascular Surgery, Faculty of Medicine, Recep Tayyip Erdogan University, Training and Research Hospital, Rize, Turkey; Department of Cardiovascular Surgery, Faculty of Medicine, Yıldırım Beyazıd University, Atatürk Training and Research Hospital, Ankara, Turkey; Department of Cardiovascular Surgery, Faculty of Medicine, Yıldırım Beyazıd University, Atatürk Training and Research Hospital, Ankara, Turkey; Department of Biomedical Engineering, Baskent University, Ankara, Turkey; Department of Biomedical Engineering, Baskent University, Ankara, Turkey; Department of Immunology, Gulhane Military Medical Academy and School of Medicine, Ankara, Turkey; Department of Anatomy, Faculty of Medicine, Hacettepe University, Ankara, Turkey

**Keywords:** cardiopulmonary bypass, oxygenator, phosphorylcholine, humoral inflammation

## Abstract

**Aim:**

To investigate and compare uncoated and phosphorylcholine-coated oxygenators in terms of induction of humoral immune response during coronary artery bypass surgery.

**Methods:**

A total of 20 consecutive patients who underwent coronary artery bypass surgery were randomly distributed into two groups according to the type of oxygenator used during surgery. Group 1 consisted of 10 patients who were operated on using phosphorylcholine-coated oxygenators. Group 2 contained 10 patients who underwent surgery using uncoated oxygenators. Blood and oxygenator fibre samples were obtained and compared in terms of immunoglobulins (IgG, IgM), complements (C3c, C4), serum total protein and albumin levels using electron microscopy and flow cytometry.

**Results:**

In group 1, levels of IgM, IgG, total protein and serum albumin were significantly increased at the end of cardiopulmonary bypass (CPB) compared to those at the beginning of CPB. In group 2, C3c and C4 levels at the beginning of CPB were found to be significantly higher than at the end. Electron microscopic examination of oxygenator fibres demonstrated that phosphorylcholine-coated fibres were less likely to be adsorbed by serum proteins and complements than the uncoated fibres.

**Conclusion:**

Our results indicate that phosphorylcholine-coated oxygenators seemed to induce humoral immune response to a lesser extent than uncoated oxygenators during coronary artery bypass procedures.

## Aim

Cardiopulmonary bypass (CPB) facilitates surgical procedures and provides adequate perfusion of other organs during cardiovascular surgery.^1^,^2^ Despite the advantages offered by CPB, a systemic inflammatory response may arise due to multiple components of the immune system, including cellular and humoral components. This inflammation may arise from contact of circulating blood cells with non-endothelial surfaces of extracorporeal circulation, as well as from ischaemia/reperfusion injury, hypothermia and other operative stresses.^1^,^2^ Cardiopulmonary and systemic hazards may occur owing to the outcomes of this inflammatory response, leading to morbidity and mortality.^1^,^3^

Modalities to manage this inflammatory response include medical agents such as steroids, complement inhibitors, monoclonal antibodies and protease inhibitors. In addition to these medications, it has been suggested that lining the inner surfaces of extracorporeal circulation systems with a relatively inert material may provide suppression of the immune response.4 The membranes of oxygenators are important in this aspect since they are directly in contact with the blood. Hence, coating these membranes is thought to aid in decreasing the inflammatory response.^4^,^5^

The objective of this study was to compare phosphorylcholinecoated and uncoated oxygenators in terms of the humoral immune response triggered during cardiopulmonary bypass surgery.

## Methods

This randomised, cross-sectional clinical study was performed in the cardiovascular surgery department of a tertiary care centre. Approval was obtained by the local institutional review board (2010/12) and all patients gave written informed consent.

(2010/12) and all patients gave written informed consent. A total of 20 consecutive patients scheduled for CPB surgery were included. During CPB, a phosphorylcholine-coated oxygenator was used in 10 patients, constituting group 1, while the uncoated oxygenator was used for the remaining 10 cases, making up group 2. Participants were allocated to the two study groups according to a computerised block-randomisation process in order to keep the number of participants in the different groups equal.

## Serum study

Complements (C3c, C4), immunoglobulins (IgG, IgM) and proteins were analysed from blood samples. A total of 5 ml of venous blood was drawn from each patient and these samples were rapidly transferred to acid–citrate–dextrose Adenin (ACD A) tubes (Becton Dickinson, Meylan, Cedex, France).

Monoclonal antibodies (20 µl) of IgG_1_FITC/IgG1PE/ PerCP were added to each tube containing 1 × 10^6^ cells. Erythrocytes were separated and removed with the addition of 2–3 ml of lysing solution (Becton Dickinson, San Jose, USA) after incubation in the dark at room temperature for 20 minutes. Subsequent to the lysing solution, the samples were irrigated with 2 ml of phosphate-buffered saline (PBS) and suspended in 500 µl PBS containing 1% paraformaldehyde.

The samples were maintained at 2–8°C in the dark until analysis. Humoral analysis was done using the FACSCanto flow cytometry system and BD FACSDiva program (Becton Dickinson, Immunocytometry Systems, San Jose, CA 95131 USA).

## Electron microscopy

Samples were gathered from the oxygenators with a sterile scalpel after opening the hard, protective cover surrounding the oxygenator with a Dremel cutting burr (Widget Supply Inc, Albany, Oregon, USA). The samples were obtained in two different sizes, containing 300 fibres (6 cm) and 50 fibres (1 cm).

Ultrasonic washing was performed on the 6-cm samples for mechanical cleaning. The fibres were maintained in 50-ml tubes containing 35 ml isotonic saline. Liquid nitrogen was added to the fibres prior to transection and electron microscopy. Electron microscopy was performed with the FEI Quanta 200 FEG scanning electron microscope (SEM) (FEI Europe, Nanoport, Eindhoven, The Netherlands) under an acceleration voltage of 22 kV.^2^

Fixation of the 1-cm fibres with 2.5% glutaraldehyde solution for 24 hours was followed by irrigation with Sorensen’s phosphate buffer (SPB). The next fixation was done with 1% osmium tetroxide, and the fibres were irrigated again with SPB solution. Increasing concentrations (25, 50, 75 and 100%) of acetone were used for dehydration.

The samples were transferred to Petri dishes and dried for six hours. After drying, the material was adhered to metallic plates of the SEM and coated with a mixture gold and palladium of 100-Å thickness using a Bio-Rad sputter apparatus (Bio-Rad Laboratories headquarters, Hercules, CA, USA). After keeping the samples in a dry medium for 24 hours, electron microscopy was performed with a Jeol SEM ASID-10 device (Jeol Ltd, Tokyo, Japan) under 80-kV acceleration voltage.

**Figure 1:**
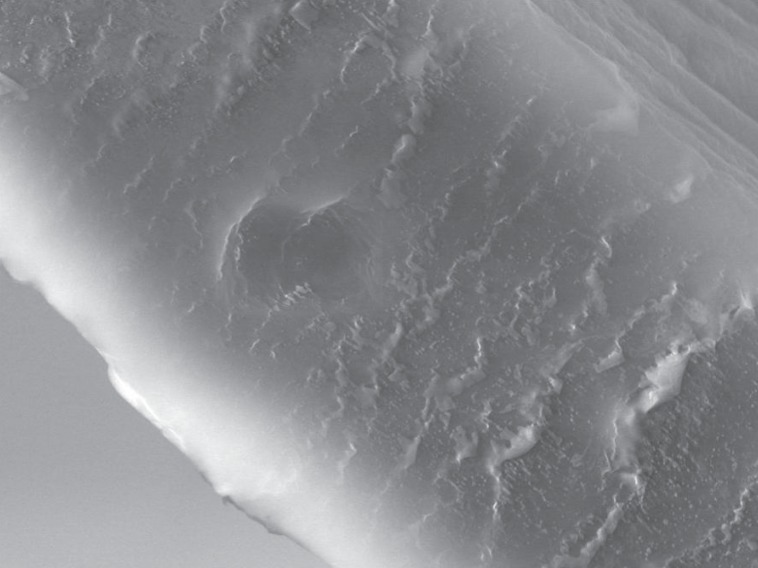
Electron microscopic view of a phosphorylcholinecoated oxygenator fibre.

**Figure 2:**
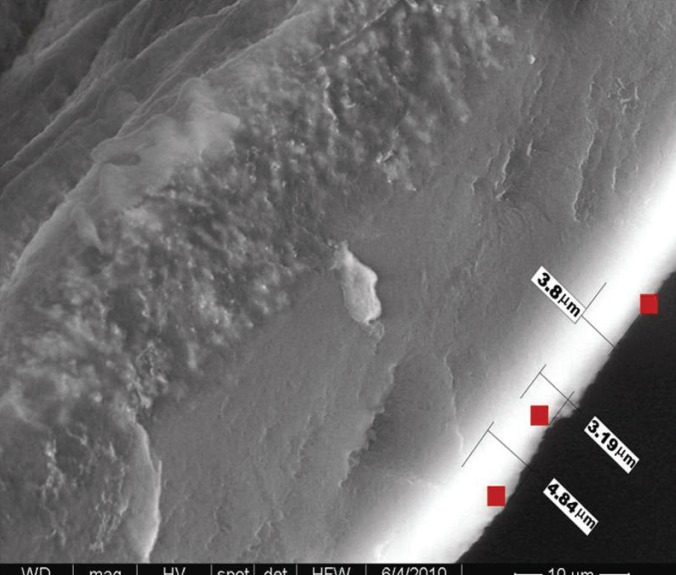
Electron microscopic view of an uncoated oxygenator fibre.

**Figure 3:**
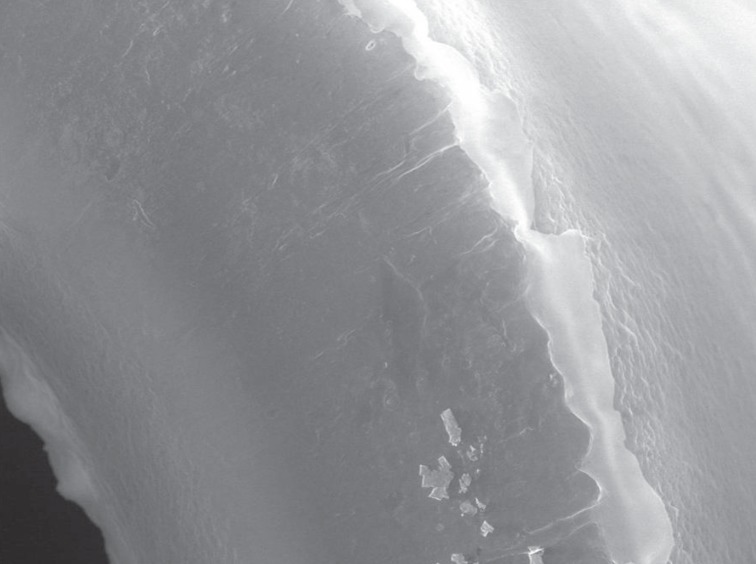
Protein adsorption on the surface of a phosphorylcholine- coated oxygenator fibre.

**Figure 4:**
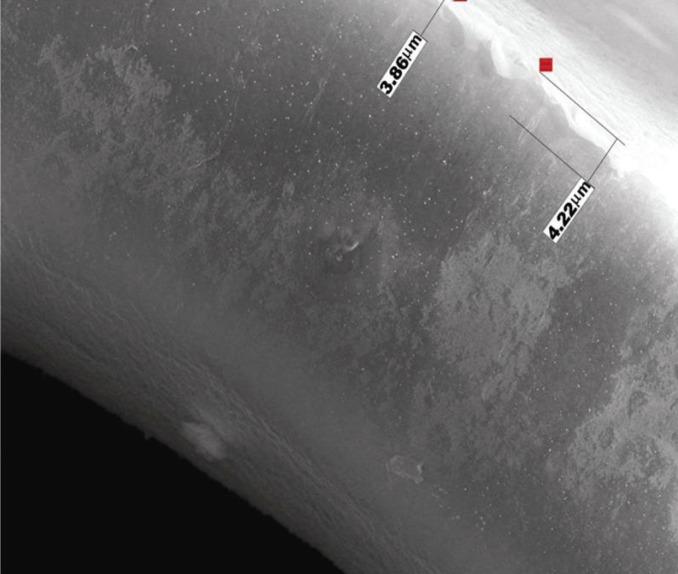
Protein adsorption on the surface of an uncoated oxygenator fibre.

Electron microscopic views of the coated and uncoated oxygenator fibres are shown in Fig. 1 and Fig. 2. Adsorption of proteins on the fibres of the coated and uncoated oxygenator fibres can be seen in Fig. 3 and 4Fig. 4.

## Statistical analysis

The Statistical Package for Social Sciences program version 16.0 (SPSS Inc, Chicago, IL, USA) was used. Descriptive data were expressed as mean, standard deviation and range (minimum to maximum values). Numbers and percentiles were used for expression of categorical variables. Parametric tests were used for data with a normal distribution, and non-parametric tests were applied to data without a normal distribution.

Distribution of normality was tested with the Kolmogorov–Smirnov test. The Mann–Whitney U- and Wilcoxon tests wereused for comparing variables between groups. Chi-squared, Fisher’s and Mantel Haenszel tests were performed for comparison of categorical variables. Level of significance was set at p < 0.05.

## Results

The study group consisted of a total of 20 patients (3 females, 17 males) with average ages of 61.7 ± 13.2 years (range, 44–78) and 63.1 ± 9.6 years (range, 51–78), for groups 1 and 2, respectively. The descriptive data and peri-operative characteristics are shown in Table 1.

**Table 1:** Descriptive and peri-operative parameters of the patients in groups 1 and 2

*Parameters*	*Group 1 (n = 10)*	*Group 2 (n = 10)*	*p-value*
Age (years),	61.7 ± 13.25	63.1 ± 9.64	0.677
median (range)	(44–78)	(51–78)	
Gender (M/F), n (%)	8/2 (80/20)	9/1 (90/10)	0.531
Risk factors			
Diabetes mellitus, n (%)	2/8 (20/80)	3/7 (30/70)	1.000
Hypertension, n (%)	6/4 (60/40)	6/4 (60/40)	1.000
COPD, n (%)	1/9 (10/90)	2/8 (20/80)	1.000
CRF, n (%)	1/9 (10/90)	2/8 (20/80)	1.000
Smoking habit, n (%)	5/5 (50/50)	7/3 (70/30)	0.361
Ejection fraction (%),	53.00 ± 3.58	47.08 ± 10.30	0.193
median (range)	(30–65)	(30–65)	
Postoperative features			
Duration of cross clamp (min),	49.60 ± 13.67	58.70 ± 23.55	0.405
median (range)	(30–77)	(40–113)	
Duration of CPB (min),	88.40 ± 26.28	102.1 ± 25.58	0.162
median (range)	(58–134)	(75–146)	
Heparin (units/ml),	4.20 ± 0.42	5.50 ± 0.84	0.001
median (range)	(4–5)	(4–7)	
Protamine (mg),	4.60 ± 0.51	5.70 ± 1.05	0.008
median (range)	(4–5)	(4–8)	
Duration of intubation (h),	8.00 ± 2.90	9.4 ± 3.74	0.488
median (range)	(4–12)	(4–18)	
ICU stay, (h),	34.00 ± 11.50	38.00 ± 11.19	0.384
median (range)	(20–48)	(24–48)	
Hospitalisation (days),	6.60 ± 0.96	8.00 ± 1.88	0.039
median (range)	(6–9)	(6–12)	
Drainage (ml),	645.00 ± 319.24	530.00 ± 182.87	0.447
median (range)	(200–1300)	(250–900)	
Transfusion (units),	920.00 ± 454.11	900.00 ± 391.57	0.934
median (range)	(600–1800)	(600–1800)	

M: male; F: female; CRF: chronic renal failure; COPD: chronic obstructive pulmonary disease; CPB: cardiopulmonary bypass; ICU: intensive care unit.

The average values for serum albumin, total protein, C3c and C4 levels were higher in group 1 at the start of the pump (Table 2). No difference was observed between the groups in terms of these variables at the end of CPB (Table 2). In group 1, total protein levels were significantly higher at the start of the pump compared to at the end of CPB (p = 0.01). A significantly lower IgG level was noted at the end of CPB compared to at the start in group 1 (p = 0.012) (Table 2).

**Table 2:** Levels of total protein, serum albumin, IgG, IgM, and complements C3c and C4.

*Variable*	*Time of measurement*	*Group 1 median (range*	*Group 2 median (range*	*p-value*
Total protein (mg/dl)	Start of pump	3.36 ± 0.31 (2.9–3.8)	4.08 ± 0.57 (3.1–4.7)	0.009^§^
	End of pump	3.82 ± 0.61 (3.1–4.8)*	4.16 ± 0.37 (3.7–4.6)	0.223
Albumin (g/dl)	Start of pump	1.96 ± 0.08 (1.9–2.1)	2.14 ± 0.31 (1.6–2.4)	0.030^§^
	End of pump	2.24 ± 0.28 (1.9–2.7)*	2.24 ± 0.23 (1.9–2.5)	0.758
IgG (g/l)	Start of pump	6.06 ± 1.71 (3.5–7.8)	7.94 ± 3.55 (3.6–12.4)	0.172
	End of pump	7.48 ± 1.96 (4.9–10.3)*	6.72 ± 2.94 (3.2–9.9)	0.448
IgM (mg/l)	Start of pump	0.48 ± 0.13 (0.43–0.72)	0.64 ± 0.27 (0.21–0.98)	0.095
	End of pump	0.54 ± 0.14 (0.39–0.73)*	0.55 ± 0.17 (0.23–0.68)	0.798
C3c (mg/dl)	Start of pump	0.58 ± 0.11 (0.4–0.7)	0.81 ± 0.19 (0.59–1.12)	0.010^§^
	End of pump	0.69 ± 0.10 (0.54–0.82)*	0.73 ± 0.07 (0.61–0.79)	0.601
C4 (mg/dl)	Start of pump	0.13 ± 0.06 (0.08–0.23)	0.16 ± 0.02 (0.13–0.19)	0.022^§^
	End of pump	0.29 ± 0.31 (0.09–0.87)	0.18 ± 0.04 (0.12–0.24)	0.315

IgG: immunoglobulin G; IgM: immunoglobulin M; C3c: complement factor C3c; C4: complement c4.

*Statistically significant difference within the same group; §statistically significant difference between the two groups.

IgM levels at the end of CPB were higher than IgM levels at the start in group 1 (p = 0.012). In group 1, mean levels of C3c were lower at the end of CPB compared to those at the start (p = 0.005) (Table 2).

## Discussion

In this study, we attempted to demonstrate whether there was a difference between the cellular immune responses of patients who underwent CPB using uncoated or phosphorylcholinecoated oxygenators. Our study indicated that a more prominent cellular immune response was observed in patients operated on using phosphorylcholine-coated oxygenators.

In spite of the advantages it offers during cardiac surgery, CPB has the potential to cause a complex inflammatory response, initiated by the contact of heparinised blood with non-endothelial surfaces.^6^ Other factors, such as bleeding, ischaemia–reperfusion injury and rejection reactions further contribute to augmentation of the immune response via secretion of vasoactive and cytotoxic cytokines, resulting in alleviation of this inflammatory cascade.^2^-^5^

Recently, modification of the surfaces that come into contact with extracorporeal circulating blood has become popular and phosphorylcholine is one of the materials used for this purpose.^7^ In the literature, there are several reports on the impact of coated oxygenators on the immune response. It was suggested that leukocyte levels increased and platelet levels decreased in both phosphorylcholine-coated and uncoated oxygenators with regard to baseline values during induction of anaesthesia.^8^,^9^

Similarly, Sohn et al. stated that there was a decrease in the postoperative platelet counts of patients who were operated on using a phosphorylcholine-coated oxygenator.^10^

Our results have shown that IgG and IgM levels were increased in group 1 at the end of CPB. This finding suggests that phosphorylcholine-coated oxygenators may induce humoral immunity. Lante et al. however found that both IgG and IgM concentrations were decreased after cardiac surgery.^11^

The complement system may be activated due to factors such as ischaemia, hypoxia, haemodilution or contact with foreign bodies.^12^ In our series, C3c levels were found to be significantly increased in group 1 at the end of CPB. Adsorption of complements to the uncoated surfaces of fibres may be an explanation for this difference. It must be remembered that even the simple circulation of blood in extracorporeal systems may lead to activation of complements.^6^

Similar to our results, de Somer et al. found no difference between uncoated and phosphorylcholine-coated systems with regard to C3 and C4 levels. The increase in C3 levels up to the first postoperative day in the phosphorylcholine-coated group was assumed to be associated with prevention of protein adsorption by the coating.^9^ Baksaas et al. found no difference with regard to levels of C3 and C4 between patients operated on using uncoated and bio-passive coated surfaces.^13^ Watanabe et al. reported that there was an increase in C3 levels in both groups in the postoperative period.^14^ In another study, comparison of phosphorylcholine- and heparin-coated oxygenators demonstrated a rise in C3 levels in both groups.^15^

Suhara et al. found thromboses on the surface of uncoated oxygenator fibres.^16^ Niimi et al. reported decreased adherence of platelets to the fibres in heparin-coated systems.^17^ However, no difference was detected with regard to protein adsorption in the same study.^17^ Gunaydin reported that less protein adsorption was observed on phosphorylcholine-coated oxygenators.^18^

The results of our study have shown that levels of albumin and total protein appeared significantly higher at the end of the operation. This increase was more in the uncoated group in our series, which is to be expected since proteins and albumin have a greater tendency to adhere to uncoated surfaces. Electron microscopy also exhibited a thicker protein layer on the surface of uncoated oxygenator fibres and this finding is in conjunction with the increased likelihood of adherence of immune system elements to uncoated surfaces.

Some limitations of this study must be noted. First, our sample size was small and strict criteria for inclusion of patients were not adhered to. Moreover, the impact of metabolic, environmental, genetic, racial and geographic factors, which could have influenced the results, could not be completely controlled. Therefore, interpretations and extrapolations must be made with caution. However, we hope that the results of this study will pioneer further trials on this topic.

## Conclusion

Despite the fact that phosphorylcholine-coated oxygenators were developed to decrease the immune response during coronary artery bypass surgery, our results have shown that a notable humoral immune response still exists with the use of these materials.
